# Supplementary material for: A Candidate for Multitopic Probes for Ligand Discovery in Dynamic Combinatorial Chemistry
Source: Molecules. 2019 Jun 8;24(11):2166. doi: 10.3390/molecules24112166 (PMC6600254; doi:10.3390/molecules24112166)
Supplement: Supplementary file 1 [file molecules-24-02166-s001.pdf]

Supporting Information

A Candidate for Multitopic Probes for Ligand Discovery in Dynamic Combinatorial Chemistry

Keiko Yoneyama, Rina Suzuki, Yusuke Kuramochi, and Akiharu Satake

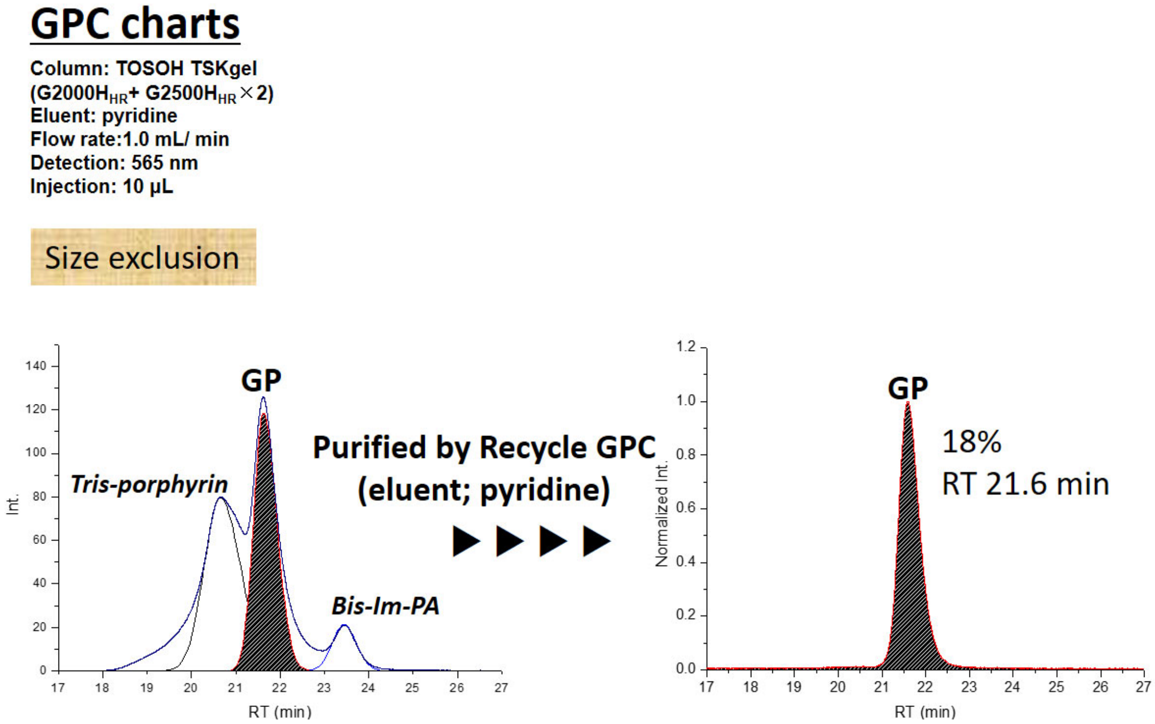

Figure S1. Analytical gel permeation chromatography (GPC) charts of **GP1Fb**. (left: a crude sample, right: purified)

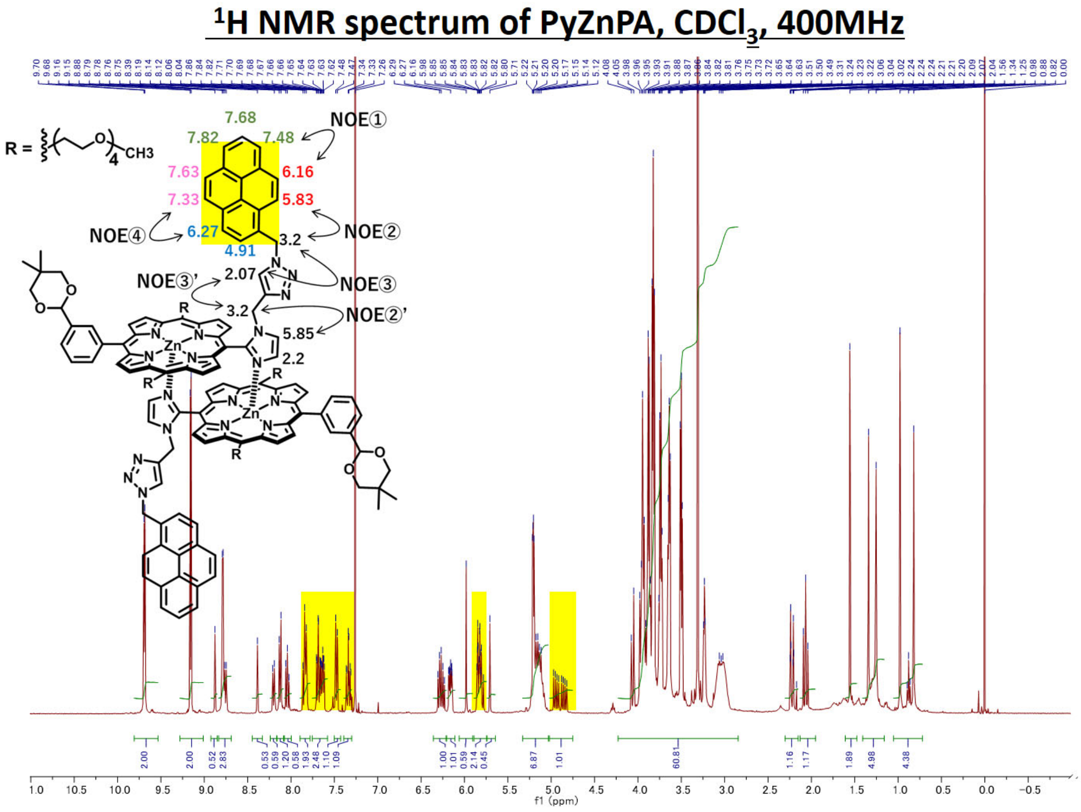

Figure S2. ^{1}H NMR spectrum (400 MHz, $CDCl_3$) of **PY-5Zn**

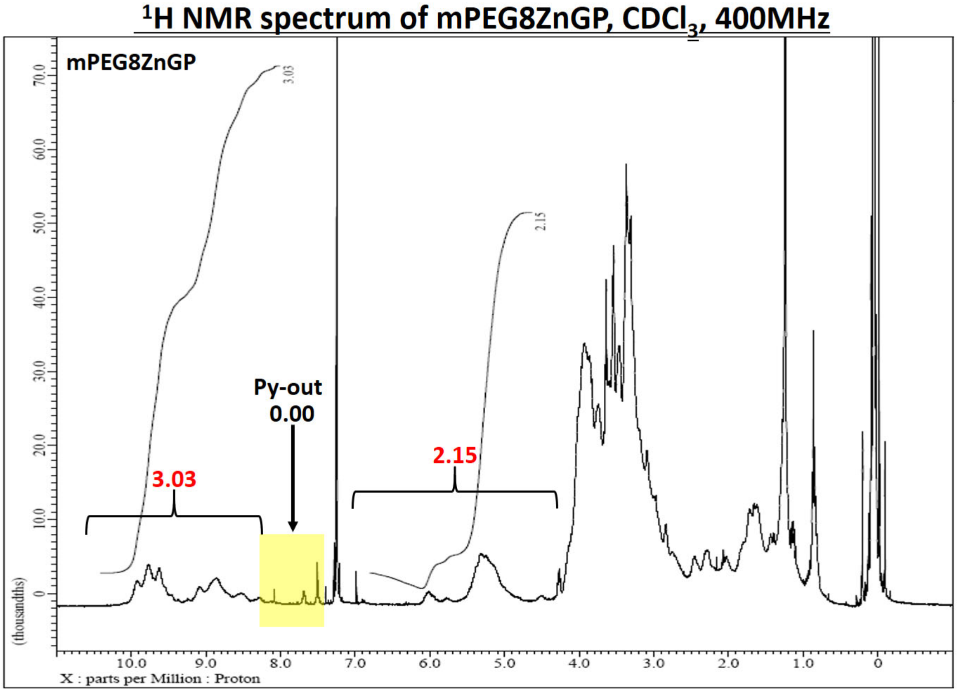

Figure S3. ^{1}H NMR spectrum (400 MHz, $CDCl_3$) of **PEG8-GP1**

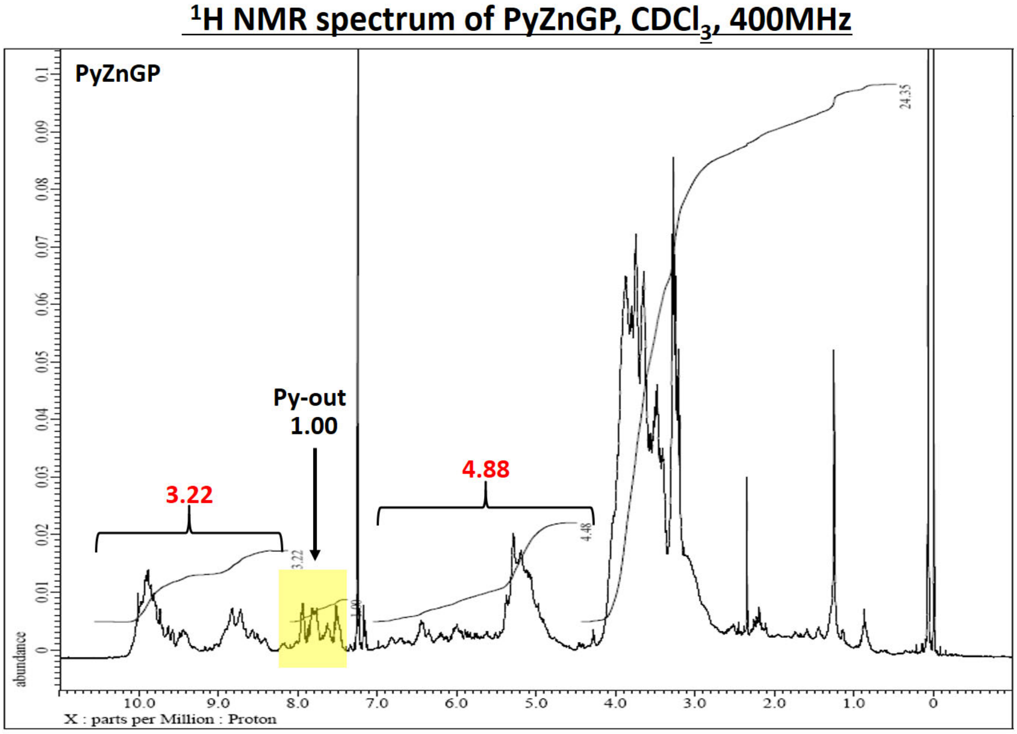

Figure S4. ^{1}H NMR spectrum (400 MHz, $CDCl_3$) of **PY-GP1**

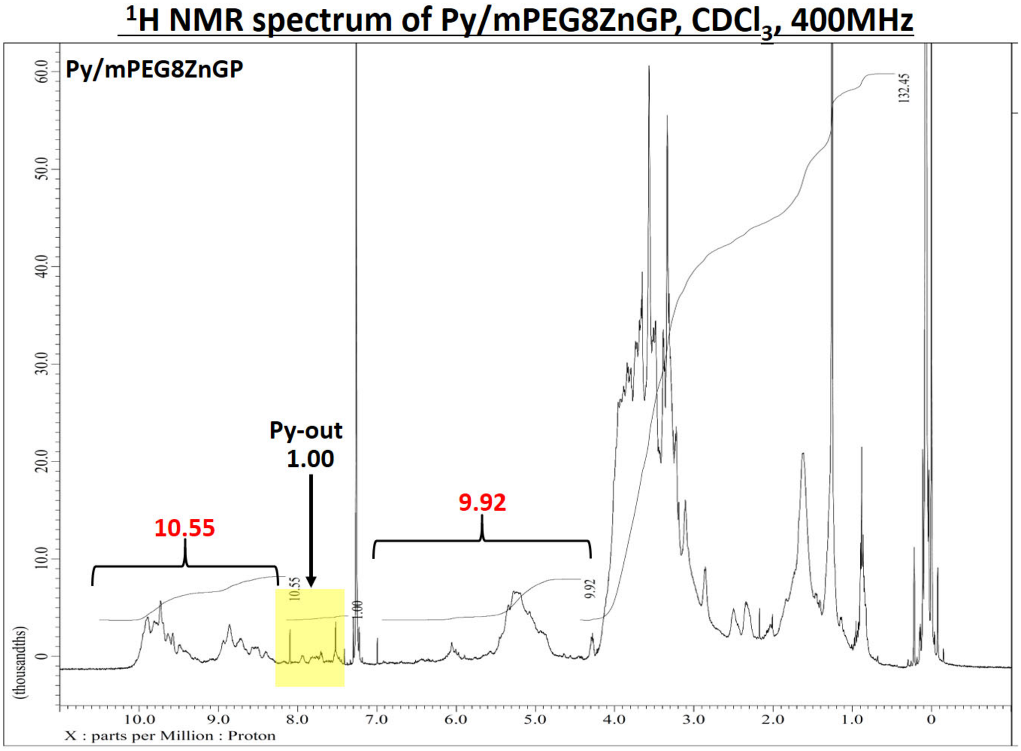

,

Figure S5. ^{1}H NMR spectrum (400 MHz, $CDCl_3$) of a reconstituted sample of **PEG8-GP 1** and **PY-GP 1** (1:1)

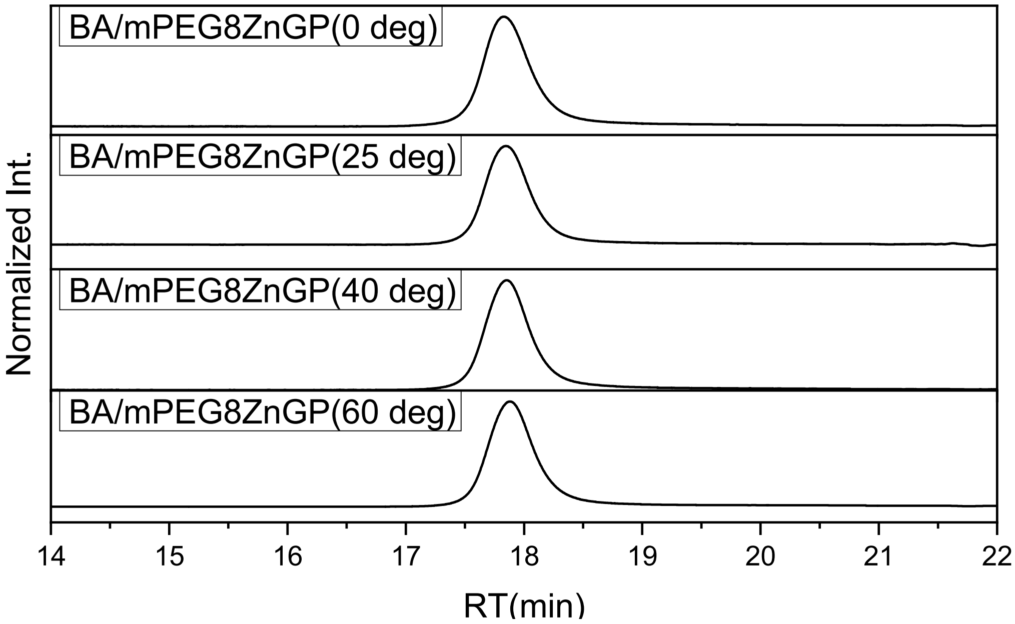

Figure S6. GPC charts of reconstituted samples of 1:1 mixture of **BA-GP1** and **PEG8-GP1**. The reconstituted temperatures are (a) 0 °C, (b) 25 °C, (c) 40 °C, and (d) 60 °C. (from top to bottom) Little change was observed among them. (column; TOSOH TSKgel G4000H_{HR}×2 (exclusion limit; 400,000 Da), eluent; $CHCl_3$:THF=95:5, flow rate; 1.0 mL/min, detection; 565 nm).

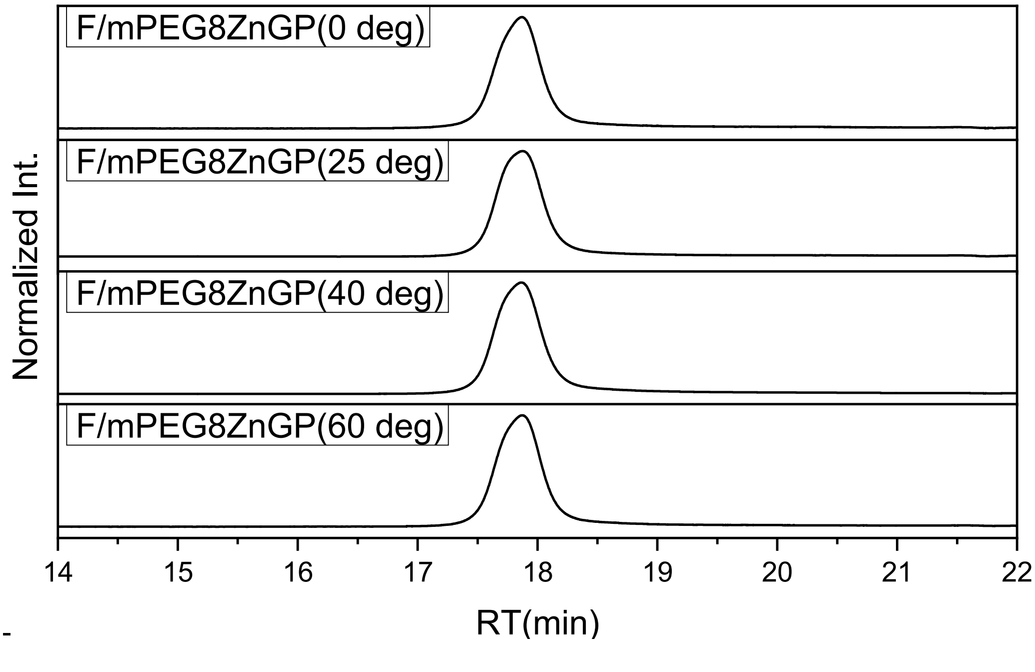

Figure S7. GPC charts of reconstituted samples of 1:1 mixture of **F9-GP1** and **PEG8-GP1**. The reconstituted temperatures are (a) 0 °C, (b) 25 °C, (c) 40 °C, and (d) 60 °C. (from top to bottom) Little change was observed among them. (column; TOSOH TSKgel G4000H_{HR}×2 (exclusion limit; 400,000 Da), eluent; $CHCl_3$:THF=95:5, flow rate; 1.0 mL/min, detection; 565 nm).

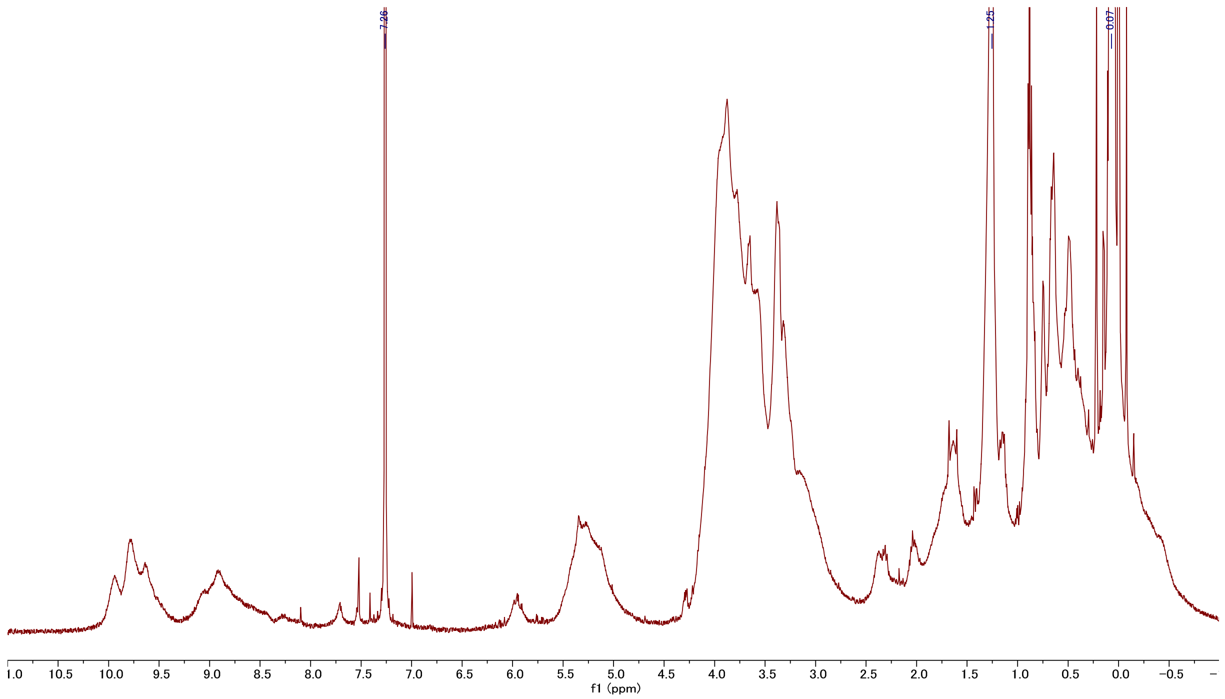

Figure S8. ^{1}H NMR spectrum (400 MHz, $CDCl_3$) of a reorganized sample of **BA-GP1**.

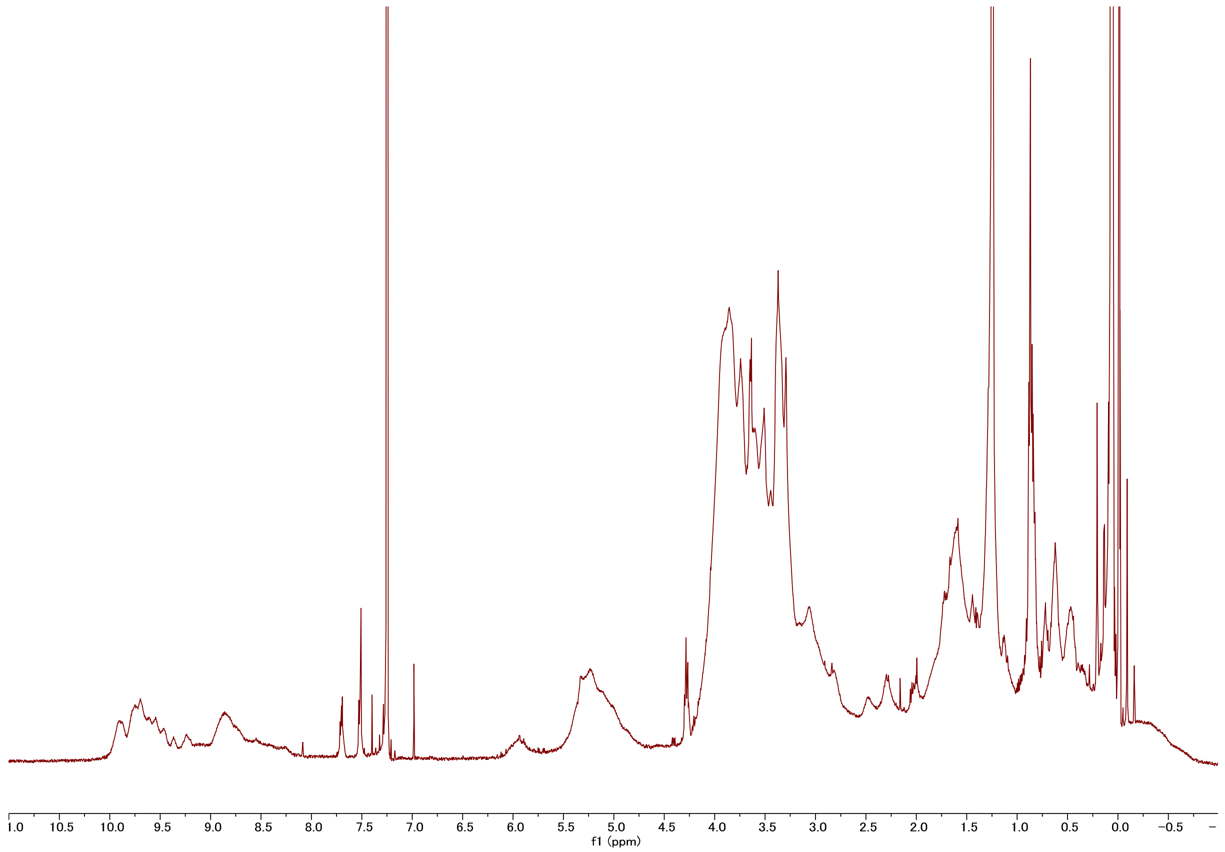

Figure S9. ^{1}H NMR spectrum (400 MHz, $CDCl_3$) of a reconstituted sample of **PEG8-GP1** and **BA-GP1** (1:1)

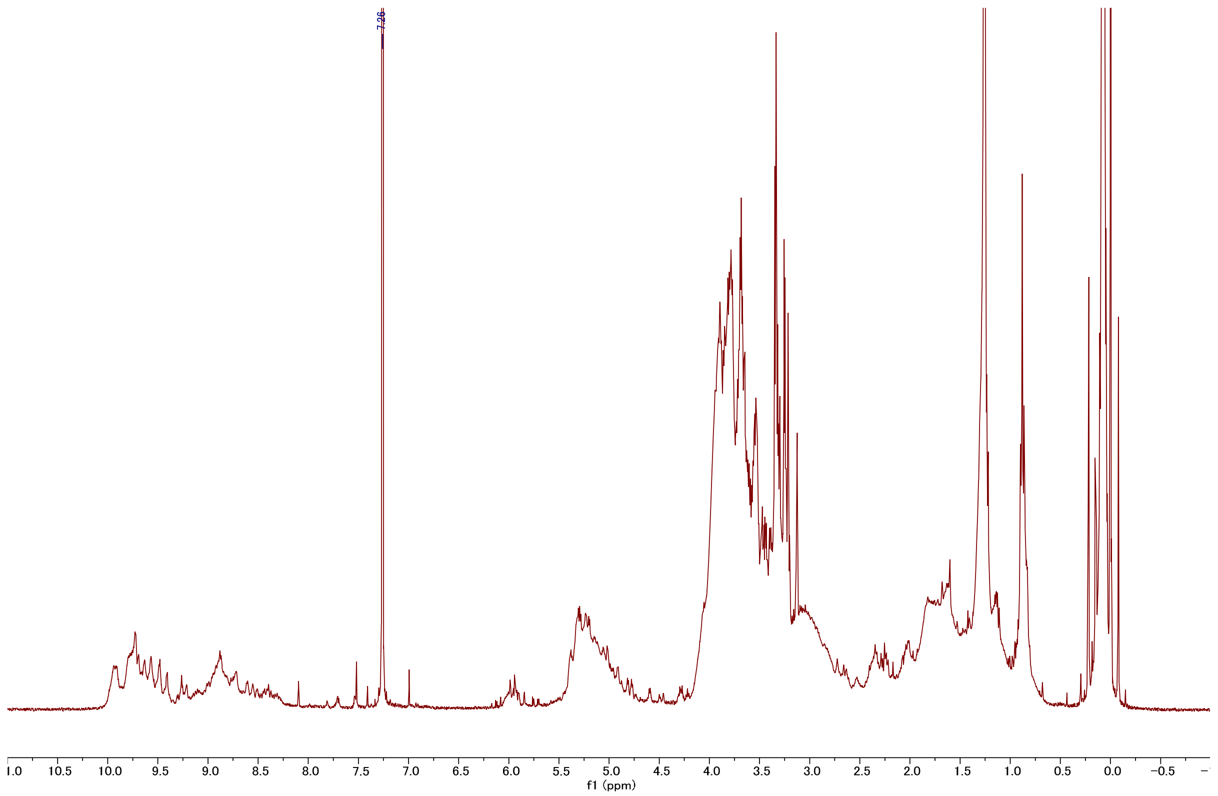

Figure S10. ^{1}H NMR spectrum (400 MHz, $CDCl_3$) of a reorganized sample of **F9-GP1**.

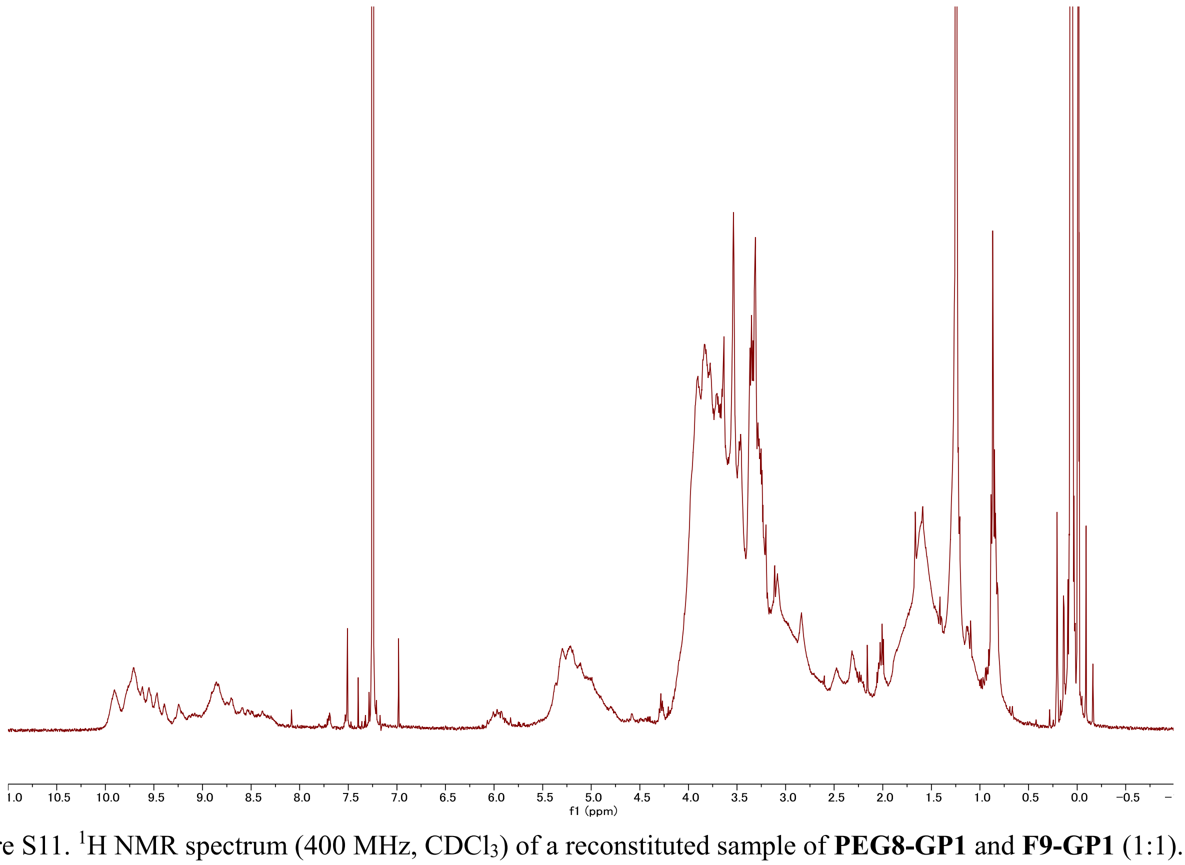

Figure S11. ^{1}H NMR spectrum (400 MHz, $CDCl_3$) of a reconstituted sample of **PEG8-GP1** and **F9-GP1** (1:1).
